# The deletion of an extra six nucleotides in the 5′ -untranslated region of the nucleoprotein gene of Newcastle disease virus NA-1 decreases virulence

**DOI:** 10.1186/s12917-014-0305-5

**Published:** 2014-12-21

**Authors:** Jiaxu Liu, Yanlong Cong, Renfu Yin, Chan Ding, Shengqing Yu, Xiufan Liu, Chunfeng Wang, Zhuang Ding

**Affiliations:** Laboratory of Infectious Diseases, College of Veterinary Medicine, Jilin University, Changchun, 130062 China; Engineering Research Center of Jilin Province for Animals Probiotics, College of Animal Science and Technology, Jilin Agricultural University, Changchun, 130118 China; Engineering Research Center of Chinese Ministry of Education for Edible and Medicinal Fungi, Jilin Agricultural University, Changchun, 130118 China; Shanghai Veterinary Research Institute, Chinese Academy of Agricultural Sciences, Shanghai, 200241 China; Animal Infectious Disease Laboratory, College of Veterinary Medicine, Yangzhou University, Yangzhou, Jiangsu 225009 China

**Keywords:** Goose, Newcastle disease virus, Reverse genetics

## Abstract

**Background:**

The virulent Newcastle disease virus (NDV) strain NA-1 (genotype VII) was isolated from an epizootic involving a flock of geese in Jilin Province, Northeast China, in 1999. Compared with the classical NDV strains, which have a genome size of 15,186 bp, the more recently isolated NDV strains, including that involved in the goose outbreak, have an extra six nucleotides in the 5′-untranslated region (UTR) of the nucleoprotein (NP) gene. This extra sequence, TCCCAC, is highly conserved and has been found in multiple NDV strains, including ZJ-1, WF00G, JSG0210, and NA-1. In the current study, an infectious clone from strain NA-1 was isolated and designated rNA-1. Subsequently, strain rNA-1 was mutated to delete the six-nucleotide insertion, producing strain rNA-1(−). Virulence of the recombinant virus was then assayed in chickens and geese.

**Results:**

The recombinant virus rNA-1(−) showed similar biological characteristics to the parental NA-1 strain in DF-1 chicken fibroblast cells. However, the virulence of rNA-1(−) in 9-day-old embryonated chicken eggs and 1-day-old specific pathogen-free (SPF) chickens was decreased compared with the rNA-1 control. Furthermore, the virulence of the recombinant strain was slightly decreased in 1-day-old SPF chickens when compared with that in 1-day-old geese.

**Conclusion:**

Following deletion of six nucleotides in the 5′-UTR of the NP gene of NDV strain NA-1, the virulence of the rNA-1(−) recombinant strain was decreased in both chickens and geese. However, rNA-1(−) was more virulent in chickens than geese, as seen by the higher intracerebral pathogenicity index value.

## Background

Newcastle disease (ND), caused by Newcastle disease virus (NDV), is one of the most serious avian diseases worldwide. NDV, also known as avian paramyxovirus serotype-1 (APMV-1), is a member of the genus *Avulavirus*, within the Paramyxoviridae family [1,2], and is a single-stranded, negative-sense, non-segmented, enveloped RNA virus [3]. The NDV genome is composed of six genes, encoding six structural proteins: nucleoprotein (NP), phosphoprotein (P), matrix (M), fusion (F), hemagglutinin-neuraminidase (HN), and RNA polymerase (L) [4]. RNA editing of P produces the additional non-structural proteins V, and possibly, W [5]. The genomic RNA associates with the NP, P, and L proteins to form the ribonucleoprotein complex, the machinery for RNA synthesis [6]. Although all NDV strains belong to a single serotype [7], there is both genetic and antigenic diversity between the strains, and the detection of progressive changes in strains isolated over successive years indicates continuous evolution [3,8].

Based on standard pathotyping assays, NDV strains are classified into three main phenotypes: velogenic, mesogenic, and lentogenic [9]. For decades, even the most virulent NDV strain was not considered to be a cause of serious disease in waterfowl. However, at least four NDV strains have been implicated in fatal diseases of geese in many provinces of South, East, and Northeast China since 1997 [10-12]. Several studies have shown that conventional live-attenuated NDV vaccines can protect birds against morbidity and mortality, but because of genotype diversity, do not prevent infection and virus shedding [13-15]. Untranslated regions (UTRs) of viral genomes play a role in the regulation of viral transcription and translation. For example, the long 3′-UTR of the M protein of measles virus and the 5′-UTR of the F protein of canine distemper virus play important roles in replication and virulence of these viruses [16,17].

Genome size in all NDV strains follows the ‘rule of six’ [18], which plays an important role in the replication of paramyxoviruses. Classical NDV strains which isolated before 1990s have a genome size of 15,186 bp [19,20]; however, NDV strains isolated recently from geese have an extra region of six highly-conserved nucleotides (TCCCAC) located in the 5′-UTR of the NP gene after 1990s in China. This sequence has been found in NDV strains such as ZJ-1 (GenBank accession no. AF431744), WF00G (GenBank accession no. FJ754273), JSG0210 (GenBank accession no. JF340367), and NA-1, the strain used in this study [21].

The current study aimed to develop a rescue system that would allow genetic modification of the virulent NA-1 strain. The system was then used to examine the effects of mutations within the 5′-UTR of the NP gene. This rescue system, based on the intracellular synthesis of T7 RNA polymerase transcripts corresponding to the NDV genome, not only serves as a technical platform for the investigation of NA-1 gene function, but may also provide an opportunity to develop a live-attenuated vaccine candidate for geese.

## Methods

### Virus, plasmids, and cells

The genotype VII NDV virus designated NA-1 was isolated from geese in Jilin Province, Northeast China, in 1999. This virus has previously been sequenced [21] and the sequence deposited in GenBank (accession no. DQ659677). Purified viruses were propagated in 10-day-old embryonated specific-pathogen-free (SPF) eggs. The helper plasmids pCI-NP, pCI-P, and pCI-L, expressing NP, P, and L, respectively, were constructed and verified as to functionality. The pBR322 plasmid, containing T7 terminator and HdvRz sequence, was constructed by our lab. Baby hamster kidney (BHK-21) cells [22], expressing the T7 RNA polymerase, and DF-1 chicken fibroblast cells were grown in Dulbecco’s modified Eagle’s medium (DMEM) containing 10% fetal calf serum.

### Birds

All birds were housed in individual ventilated cages supplied with filtered air under a 12-h light/dark cycle. SPF hygiene status was approved by Harbin Veterinary Research Institute. Food and water were available *ad libitum*.

### Construction of a full-length NA-1 cDNA clone

**Figure 1:**
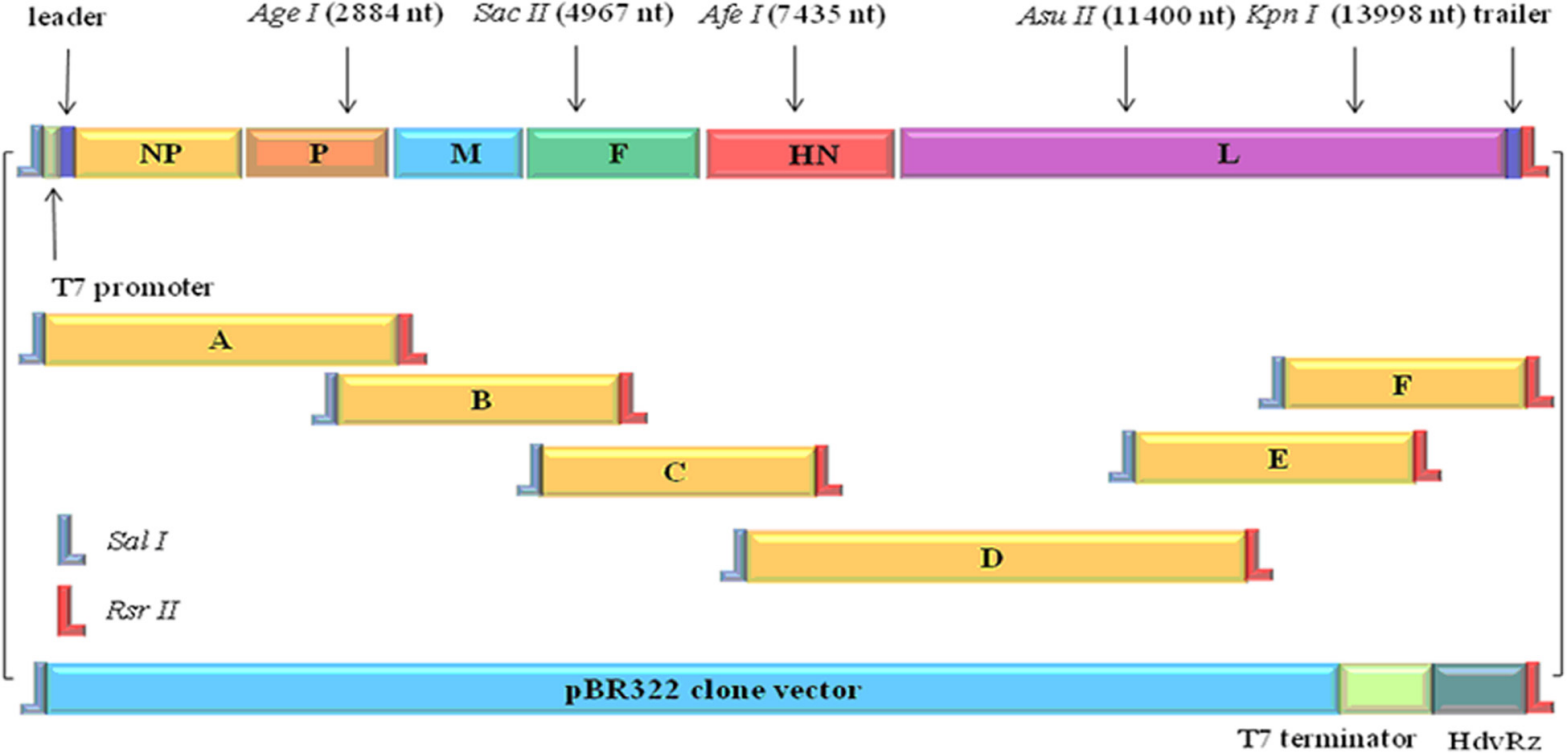
**Full-length cDNA construction strategy.** Segment A contained the T7 promoter, and three G nucleotides were inserted between the leader sequence and the T7 promoter to enhance the rescue efficiency of virus.

RNA from NA-1 was extracted from allantoic fluid using TRIzol (Invitrogen) according to the manufacturer’s instructions. cDNA was prepared using a Superscript II reverse transcriptase kit (Invitrogen) and random hexamers, as per the manufacturer’s instructions. Six pairs of specific primers (sequences available on request) were designed to amplify the complete genomic cDNA complement. Resulting PCR amplicons, named A, B, C, D, E, and F, were purified and cloned into pMD18-T vector (TaKaRa). A full-length cDNA copy of the NA-1 genome was constructed using a modified pBR322 cloning vector (Figure 1), and designated pAF. Six segments of the NA-1 genome were amplified from pAF and sequenced to verify accurate assembly.

There is one RsrII site in the F protein segment of the NA-1 genome. To differentiate rescue virus from wild-type, the interior RsrII site was synonymously mutated from cggtc*c*g to cggtc*t*g using overlap PCR with primers 6FMP3 (5′-CCTCAGCCTCCATGTAAATCAC*a*GACCGAATGACTGT-3′) and 6FMP4 (5′-CACATTGACACAGTCATTCGGTC*t*GTGATTTAC-3′). Another pair of primers, 6FP1 (5′-CGCGTCGACTCAGAGAACGAGAGGACAGAGATAC-3′) and 6FP2 (5′-CGCCGGTCCGACCAAACAGAGATTTGGTGAATG-3′), were designed to amplify the F segment of the NA-1 genome in a two-step process. The first part of the F segment was amplified using 6FP1 and 6FMP3 at 54°C. Then, primers 6FMP4 and 6FP2 were used to amplify the second part at 55°C.

### Construction of a full-length cDNA clone with 5′-UTR deletion

The 5′-UTR of the NP gene was located in PCR fragment A described above. Using the plasmid constructed from segment A and the pMD18-T vector (named T-A), the six additional nucleotides (nucleotides 1648–1653) in the 5′-UTR of the NP gene were deleted using PCR. Primer pair 6AMP1 (5′-TCCGCCCAAAACCCACTCCCCGACCC-3′) and 6AMP2 (5′-GGGTCGGGGAGTGGGTTTTGGGCGGA-3′) was designed to amplify T-A and delete the six nucleotides. The resulting PCR product was digested using DpnI. Plasmids were transformed into competent cells DH5α, and clones containing the 6-bp deletion (named T-A(−)) were screened by PCR. To construct a full-length cDNA clone, T-A(−) was used to replace the same segment in pAF using SalI and AgeI. The resulting product was named pAF(−). Six segments of rNA-1 were amplified from pAF(−) and sequenced.

### Recovery and identification of infectious NDV from cDNA

BHK-21 cells were grown overnight to 70–80% confluence in six-well culture dishes. These were cotransfected with 0.25 μg of pAF or pAF(−), 0.4 μg of pCI-NP, 0.2 μg of pCI-P, and 0.2 μg of pCI-L, using 8 μL of X-tremeGENE HP DNA Transfection Reagent (Roche). pAF, pAF(−), pCI-NP, pCI-P, and pCI-L were transfected into BHK-21 cells using liposome method separately as controls. Following incubation for 6 h, the culture medium was replaced with complete DMEM medium containing 5% allantoic fluid. After 5 days, the supernatant was harvested, passed through a 0.22 μm filter, and inoculated into the allantoic cavities of 10-day-old embryonated SPF eggs. Allantoic fluid was harvested after 96 h and hemagglutination assays (HA) were performed according to the World Organization for Animal Health’s standard method [23]. Viral RNA was extracted from allantoic fluid and the F segment was amplified, cloned, and digested with RsrII (as described above) for identification.

### Growth curve

To generate a growth curve, replication of the parental and rescued viruses was examined in DF-1 cells infected at a multiplicity of infection of 10 under one-step growth conditions. After adsorption for 1 h, the DF-1 cell monolayer was incubated in DMEM supplemented with 5% fetal calf serum at 37°C and 5% CO_2_. At 8-h intervals, the supernatant was collected to determine viral titers in a 50% tissue culture infection dose (TCID_50_) per milliliter by end-point titration. All titrations were performed in triplicate.

### Genetic stability and virulence

The rescued virus was passaged in 9-day-old SPF eggs, obtained from the Harbin Veterinary Research Institute, to assess the genetic stability and pathogenicity of the rescued virus *in vivo*. After 3 days, the allantoic fluids were examined by HA assay [24] to confirm effective viral replication. The virus, at a 2^9^ HA titer, was diluted by 0.001% every passage and the inoculated into the allantoic cavity of three 10-day-old embryonated SPF eggs at 37°C. Each virus generation was identified using reverse-transcription polymerase chain reaction (RT-PCR), and the 56–1789 nucleotide region of the NP gene was sequenced after each passage of NA-1. After 10 passages, the mean time until death (MDT) and intracerebral pathogenicity index (ICPI) were determined in embryonated SPF eggs or in SPF chickens [25].

### Quantitative real-time PCR and western blot

The total RNA of each of the virus strains was extracted using TRIZOL reagent (Invitrogen), and cDNA was subsequently synthesized using a random hexamers primer, and then standardized to 1000 ng of total RNA per sample. To assess the transcription level of the NP gene, gene-specific primers were designed. The primers used for these mRNA analyses were: 5′-TGTACCACCAACTGCTTG-3′ and 5′-ATCACGCCACAGTTTCC-3′ for GAPDH, 5′-GGCAGCATGGACATTCCT-3′ and 5′- CCTTGCGGCTTGTTTGAT-3′ for NP. Each real-time PCR reaction (20 μL volume) contained 10 μL SYBR Green real-time PCR master mix (TAKARA), 0.25 μM gene-specific primers, and 1 μL standardized template cDNA. Amplification conditions were as follows: 95°C for 5 min, followed by 35 cycles of 95°C for 30 s, 56°C for 30 s, and 72°C for 30 s. After a final extension at 72°C for 10 min, a melting-curve analysis was performed to ensure specificity of the products. NP gene transcription levels were normalized to that of GAPDH, and transcription relative to the sample with the lowest expression was calculated using the 2^-ΔΔCT^ method [26].

To assess the effects of deletions in the UTR of the NP gene on translation, NP protein expression by the NDV mutant was analyzed by western blotting [27]. DF-1 cells were infected at 5 PFU/cell, and total proteins were collected at 24 h post-infection. Cells were washed with PBS, scraped, collected by low-speed centrifugation, and lysed with lysis buffer (6.25 mM Tris [pH 6.8], 1% SDS, 10% glycerol, 6 M urea, 0.01% bromophenol blue, 0.01% phenol red) for 30 min on ice. The lysates were clarified by centrifugation at 7,000 × *g* for 10 min and used for western blot analysis. Samples of the cell lysate were mixed with Laemmli sample buffer and boiled for 3 min prior to electrophoresis. The boiled samples were separated by SDS-polyacrylamide gel electrophoresis on a 12% gel, and the resolved proteins were transferred to nitrocellulose membrane and incubated using a primary monoclonal antibody against the NDV NP protein, or GADPH protein as a loading control. The membranes were washed with PBS-T, and then incubated with the second horseradish peroxidase-conjugated goat anti-rabbit IgG antibody. Bands were visualized using SuperSignal West Pico Substrate (Thermo Scientific). This experiment was performed in triplicate.

### Experiment in geese

One-day-old Huoyan geese, obtained from in Jilin Province, Northeast China, were negative for both NDV antigen and maternal antibody. Thirty 1-day-old geese were divided into three equal groups. Purified NA-1 virus, with a hemagglutination assay (HA) titer of 2^9^, was diluted 1/10 in sterile isotonic saline, and 0.05 mL of the diluted virus was injected intracerebrally into each of 10 geese in group 1. Groups 2 and 3 were infected with rNA-1 (HA titer 2^9^) and rNA-1(−) (HA titer 2^9^), respectively, using the same method. The birds were observed for clinical symptoms and mortality daily for 8 days. At each time point the birds were scored as follows: 0, no clinical symptoms; 1, showing signs of illness, and 2, deceased. The intracerebral pathogenicity index (ICPI) results represent the mean score per bird per observation over the 8-day period.

### Statistical analyses

All values are the mean ± SEM of at least three individual samples for the *in vitro* experiments. We used analysis of variance, as calculated by GraphPad Prism 5, to establish the statistical significance of differences between the experimental groups. Individual inter-group comparisons were made using a two-tailed unpaired t-test with Welch’s correction. Differences were considered significant at *P* < 0.05 (*).

### Ethics statement

Animals were treated humanely and with regard for alleviation of suffering. All animal experiments were carried out according to the experimental practices and standards approved by the Animal Welfare and Research Ethics Committee at Jilin University (Approval ID: 20130713–1).

## Results

### Identification of full-length cDNA

To determine whether the cDNA segments combined in our modified pBR322 vector (pAF from NA-1 and pAF(−) from NA-1 minus the additional nucleotides in the NP 5′-UTR) were full-length copies of the viral genome, six segments of pAF and pAF(−) were amplified and sequenced. Sequencing showed that pAF and pAF(−) were full-length cDNA copies of the parental virus. pAF(−) was identical to pAF except for the 6-bp deletion in the 5′-UTR of the NP gene at nucleotides 1648–1653.

### Characterization of the rescued virus

To generate infectious NDV from the cDNA of pAF and pAF(−), plasmids were cotransfected with BHK-21 cells and incubated for 5 days. On day 6, the supernatants from the experimental and control groups were inoculated into the allantoic cavities of SPF 10-day-old embryonated eggs. Allantoic fluid was harvested 96 h later and virus identified via HA assay. The viruses rescued from pAF and pAF(−) were named rNA-1 and rNA-1(−), respectively, and had identical genome sequences apart from the 6-bp deletion in the 5′-UTR of the NP gene. The rescued rNA-1 and rNA-1(−) viruses had HA titers of 2^4^ and 2^3^, respectively.

**Figure 2:**
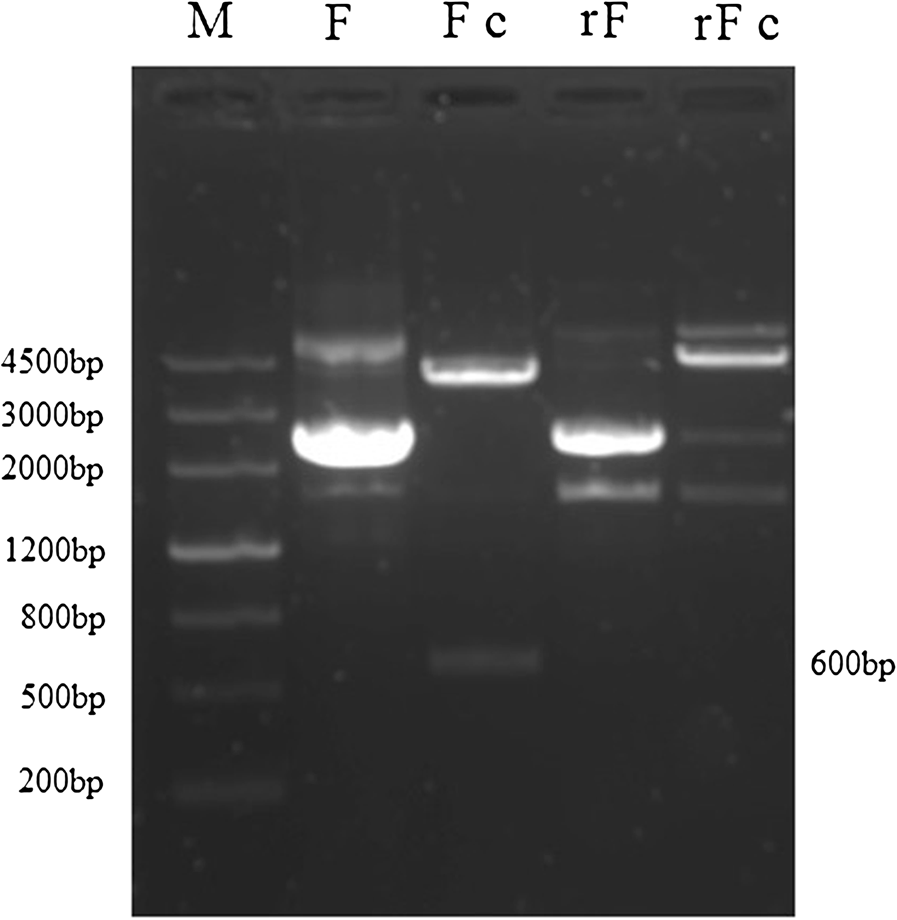
**F segment from NA-1 and rNA-1 viruses.** Lane 1: marker, lane 2: F segment from NA-1 virus cloned into pMD18-T vector, lane 3: NA-1 F segment digested by RsrII, Lane 4: F segment from rNA-1 virus cloned into pMD18-T vector, lane 5: F segment from rNA-1 digested by RsrII.

F segments of rNA-1 and NA-1 that were amplified by 6FP1 and 6FP2, respectively, and digested with RsrII showed that rNA-1 can be differentiated from parental virus NA-1 by restriction fragment length polymorphism (Figure 2).

### Transcription and translation of NP

**Figure 3:**
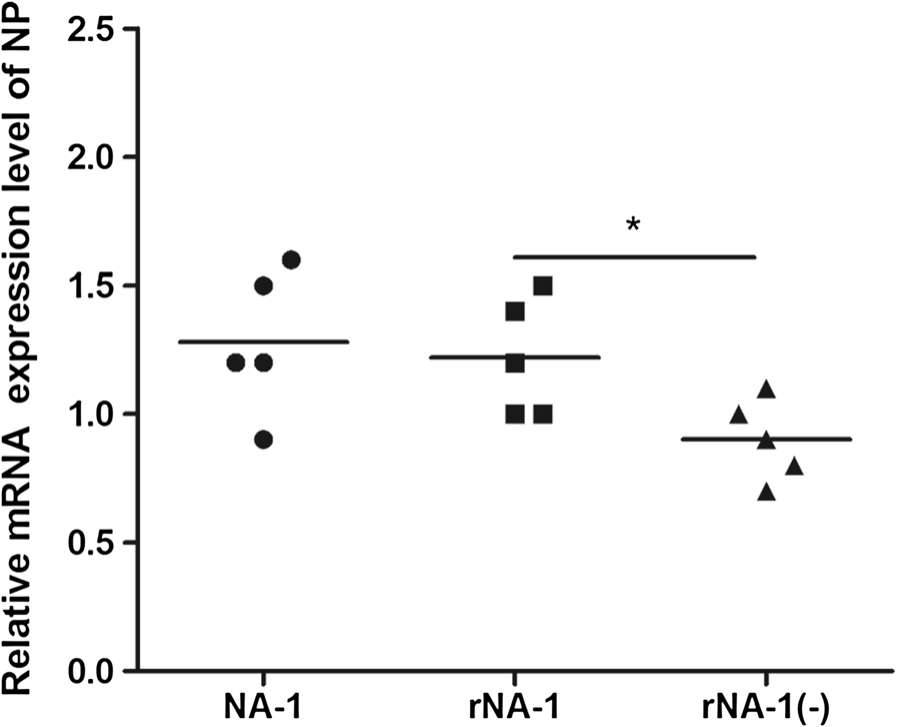
Relative mRNA expression of the NP gene in DF1 cells infected with recombinant viruses (*, p < 0.05).

**Figure 4:**
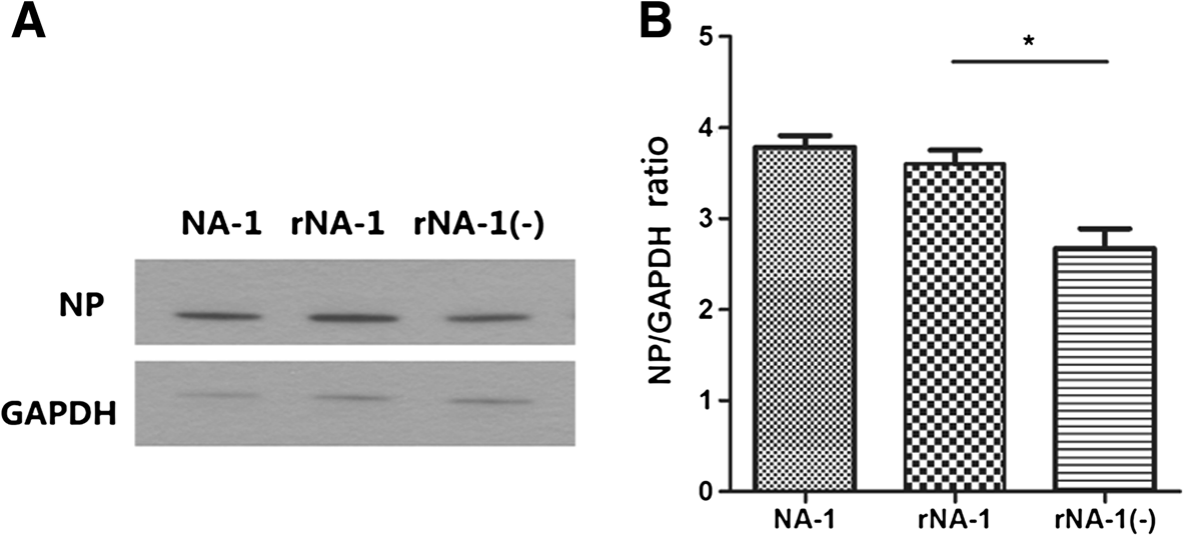
**Protein synthesis in DF-1 cells infected with recombinant viruses. A.** Western blot of NP and GAPDH proteins from rNA-1(−), rNA-1, and NA-1 virus-infected cells. **B.** Ratios of quantified NP protein to GAPDH protein levels from the rNA-1(−), rNA-1, and NA-1 strains (*, p < 0.05). Each bar shows the mean and standard error of the mean of triplicate samples.

Quantitative real-time PCR analysis showed that expression of NP was significantly lower in rNA-1(−) than in rNA-1 and NA-1 (*P* < 0.05), and that there was no significant difference in expression levels between NA-1 and rNA-1 (*P* > 0.05) (Figure 3). Western blot analysis confirmed that there was no significant difference in the ratio of NP to GAPDH between NA-1 and rNA-1 (*P* > 0.05), but that the ratio in rNA-1(−) was significantly lower than that of rNA-1 or NA-1 (*P* < 0.05) (Figure 4). This result indicates that the 6-bp deletion in the 5′-UTR of the NP gene at nucleotides 1648–1653 decreases the expression of the NP gene.

### Growth and virulence analysis

**Figure 5:**
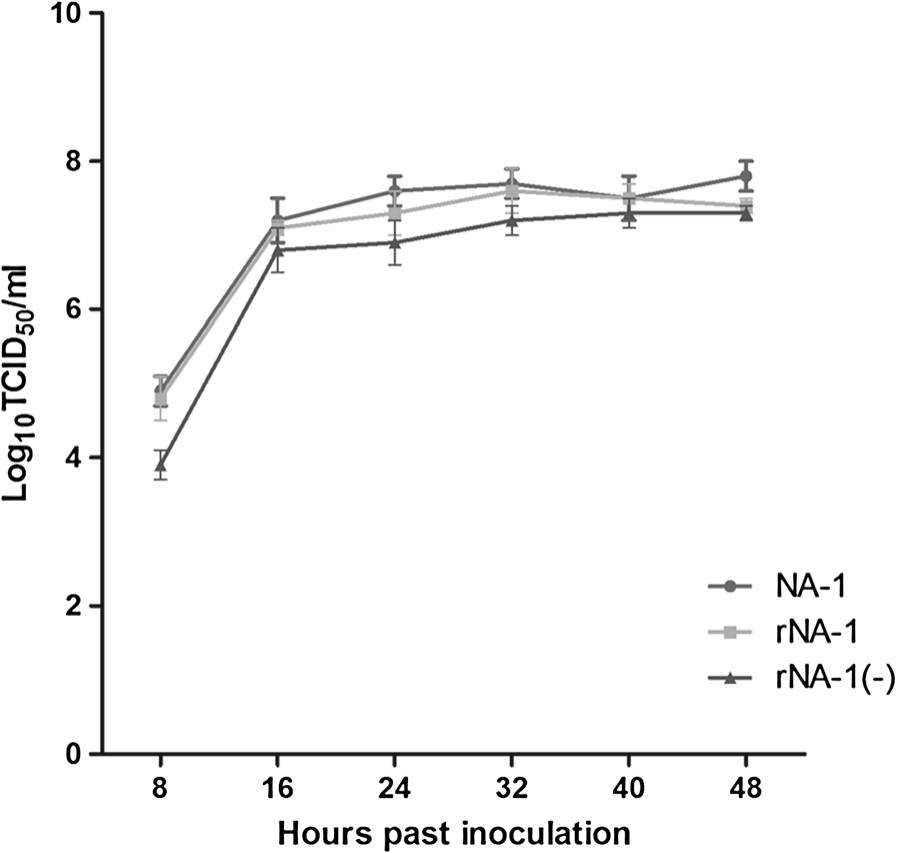
**Single-step growth curves of NA-1, NA-1, and rNA-1(−) in DF-1 cells.**

Growth curves showed that rNA-1(−) replicated more slowly than rNA-1, but that the two strains have similar growth characteristics (Figure 5). Sequencing and alignment of rNA-1 and rNA-1(−) during serial passages in 10-day-old embryonated SPF eggs showed no genetic drift during the 10 separate passages. After the final passage, two standard pathogenicity tests, MDT and ICPI, were performed to compare the virulence of rNA-1, rNA-1(−), and NA-1. The MDT and ICPI of wild-type NA-1 have previously been determined to be 59.6 h and 1.65, respectively. It is generally agreed that NDVs with ICPI values greater than 1.5 are categorized as velogenic strains. The MDT and ICPI values for rNA-1 from the 10^th^ passage were 59 h and 1.62, respectively. The MDT and ICPI values for rNA-1(−) from the 10^th^ passage were 62 h and 1.52, respectively (Table 1). These data demonstrated that strains rNA-1 and rNA-1(−) remained velogenic and genetically stable during serial passages *in vivo*. Although the virulence of rNA-1(−) did not change significantly in chickens, it was decreased compared with NA-1 and rNA-1.

**Table 1:** **Pathological characteristics of NA-1, rNA-1, and rNA-1(−)**

**Virus**	**MDT** ^**a**^	**ICPI** ^**b**^ **from chicken**	**ICPI** ^**b**^ **from goose**
NA-1	59.6 h	1.65	1.60
rNA-1	59 h	1.62	1.60
rNA-1(−)	62 h	1.52	1.35

^a^Lentogenic strains, MDT > 90 h; mesogenic strains, MDT = 60–90 h; velogenic strains, MDT < 60 h.

^b^Lentogenic strains, ICPI < 0.7; mesogenic strains, ICPI = 0.7–1.5; velogenic strains, ICPI = 1.5–2.0.

### Experiment in geese

The pathogenicity of the viruses in geese was examined, and the results were calculated by the ICPI method. At 10 days post-infection, all animals from the three groups were dead. The ICPI results for NA-1 and rNA-1 were 1.60, while the value for rNA-1(−) was 1.35. This indicated that rNA-1(−) was less pathogenic than rNA-1 in geese.

## Discussion

Highly pathogenic NDV causes a rapidly spreading illness in poultry, with four worldwide pandemics since its emergence in the early 20^th^ century. The most recent pandemic was caused by genotypes VII and VIII in the late 1980s and 1990s [28,29]. For decades, this virus was not considered to be infectious in mammals and waterfowl. However, cases of waterfowl infection have been frequent in China since the late 1990s. According to recent reports, virulent strains isolated from geese were responsible for outbreaks of ND in waterfowl and belonged to genotype VII [30,31]. It is still unclear whether NDVs circulated amongst chickens have the potential to become highly pathogenic in waterfowl, or whether the specificity of receptors could influence the host range of the virus and viral virulence.

Virulent NDV strains are still isolated frequently from vaccinated birds, demonstrating that NDV remains a sustained threat to commercial flocks. Nowadays, the prevailing NDV strain of geese in China is genotype VII, while vaccine strains such as B1, Clone30, La Sota, and V4, which are used to protect geese from NDV infection, belong to different genotypes. The antigenic differences between the prevailing and vaccine strains might explain current ND outbreaks in vaccinated poultry flocks [15,32,33].

Methods for controlling and preventing ND may be the same as those used for avian influenza virus, with a significant need for epidemiological surveillance. The best control method is the development of homologous vaccines with high efficiency and safety. However, no current commercial vaccine is available for geese for protection against virulent genotype VII NDV. Therefore, we set out to establish a reverse genetics system that allows the genetic manipulation of a virulent strain, which could be used as a vaccine candidate or a viral vector to express foreign genes in our ongoing studies.

The amino acid sequence at the F protein cleavage site is a major determinant of NDV virulence [34,35], although other genes also have an effect on virulence [36]. Transcriptional and translational control signals may also modulate virulence by controlling protein expression, as has been described for canine distemper virus and NDV [16,37,38]. A Typically ICPI is only performed in one day old SPF chickens. However, due to tropism differences that has been observed with different NDV strains in different poultry species, that an ICPI was warranted in geese. That the geese were not SPF due to lack of availability, but that they were NDV antibody negative. The rNA-1 and rNA-1(−) viruses had HA titers of 2^4^ and 2^3^, respectively, in the current study. A correlation between virulence and the efficiency of viral replication has been observed for many viruses [36]. The lower titer of the rNA-1(−) strain may be the result of the decreased levels of transcription and expression of the NP gene following deletion of the six nucleotides from the 5′-UTR. This might also account for the low ICPI value in geese. We must note that the contribution of genes to virulence may be dependent on the particular virus strain used. Additionally, because of the selective pressure from the immune system, the virus can evolve by itself. Therefore, it is not the best way to modify the HN and F genes of velogenic NDV for obtaining live-attenuated vaccine; the greater the pressure, the greater the resistance.

## Conclusions

The results of this study demonstrate that the virulence of the rNA-1(−) strain is still at the edge of the velogenic pathotype, and is not significantly different from that of the rNA-1 strain. However, the pathogenicity of rNA-1(−) towards geese is lower than that of rNA-1. Therefore, the deletion of the six nucleotides from the 5′-UTR of the NP gene could contribute to the decrease in virulence of the mutant NA-1 strain.
